# A Nonlinear Relation between Body Mass Index and Long-Term Poststroke Functional Outcome—The Importance of Insulin Resistance, Inflammation, and Insulin-like Growth Factor-Binding Protein-1

**DOI:** 10.3390/ijms25094931

**Published:** 2024-04-30

**Authors:** Gustaf Gadd, Daniel Åberg, Alexander Wall, Henrik Zetterberg, Kaj Blennow, Katarina Jood, Christina Jern, Jörgen Isgaard, Johan Svensson, N. David Åberg

**Affiliations:** 1 Department of Internal Medicine and Clinical Nutrition, Institute of Medicine, The Sahlgrenska Academy, University of Gothenburg, 405 30 Gothenburg, Sweden; daniel.aberg@medic.gu.se (D.Å.); alexander.wall@vgregion.se (A.W.); jorgen.isgaard@medic.gu.se (J.I.); johan.svensson@medic.gu.se (J.S.); david.aberg@medic.gu.se (N.D.Å.); 2 Region Västra Götaland, Department of Acute Medicine and Geriatrics, Sahlgrenska University Hospital, 413 45 Gothenburg, Sweden; 3 Region Västra Götaland, Department of Specialist Medicine, Sahlgrenska University Hospital, 413 45 Gothenburg, Sweden; 4 Närhälsan, Region Västra Götaland, 411 04 Gothenburg, Sweden; 5 Clinical Neurochemistry Laboratory, Sahlgrenska University Hospital, 431 80 Mölndal, Sweden; 6 Department of Psychiatry and Neurochemistry, Institute of Neuroscience and Physiology, Sahlgrenska Academy, University of Gothenburg, 431 41 Mölndal, Sweden; 7 Department of Neurodegenerative Disease, UCL Queen Square Institute of Neurology, University College London, London WC1E 6BT, UK; 8 Hong Kong Center for Neurodegenerative Diseases, Hong Kong, China; 9 Wisconsin Alzheimer’s Disease Research Center, School of Medicine and Public Health, University of Wisconsin-Madison, Madison, WI 53707, USA; 10 UK Dementia Research Institute, University College London (UCL), London WC1E 6BT, UK; 11 Paris Brain Institute, ICM, Pitié-Salpêtrière Hospital, Sorbonne University, 75005 Paris, France; 12 Neurodegenerative Disorder Research Center, Division of Life Sciences and Medicine, Department of Neurology, Institute on Aging and Brain Disorders, University of Science and Technology of China and First Affiliated Hospital of USTC, Hefei 230001, China; 13 Department of Clinical Neuroscience, Institute of Neuroscience and Physiology, The Sahlgrenska Academy, University of Gothenburg, 405 30 Gothenburg, Sweden; katarina.jood@neuro.gu.se; 14 Region Västra Götaland, Department of Neurology, Sahlgrenska University Hospital, 413 45 Gothenburg, Sweden; 15 Institute of Biomedicine, Department of Laboratory Medicine, Sahlgrenska Academy, University of Gothenburg, 405 30 Gothenburg, Sweden; 16 Region Västra Götaland, Department of Clinical Genetics and Genomics, Sahlgrenska University Hospital, 413 45 Gothenburg, Sweden; 17 Region Västra Götaland, Department of Internal Medicine, Skaraborg Central Hospital, 549 49 Skövde, Sweden

**Keywords:** obesity paradox, insulin-like growth factor-binding protein-1 (IGFBP-1), body mass index (BMI), modified Rankin Score (mRS), homeostasis model assessment of insulin resistance (HOMA-IR)

## Abstract

Both high serum insulin-like growth factor-binding protein-1 (s-IGFBP-1) and insulin resistance (IR) are associated with poor functional outcome poststroke, whereas overweight body mass index (BMI; 25–30) is related to fewer deaths and favorable functional outcome in a phenomenon labeled “the obesity paradox”. Furthermore, IGFBP-1 is inversely related to BMI, in contrast to the linear relation between IR and BMI. Here, we investigated s-IGFBP-1 and IR concerning BMI and 7-year poststroke functional outcome. We included 451 stroke patients from the Sahlgrenska Study on Ischemic Stroke (SAHLSIS) with baseline measurements of s-IGFBP1, homeostasis model assessment of IR (HOMA-IR), BMI (categories: normal-weight (8.5–25), overweight (25–30), and obesity (>30)), and high-sensitivity C-reactive protein (hs-CRP) as a measure of general inflammation. Associations with poor functional outcome (modified Rankin scale [mRS] score: 3–6) after 7 years were evaluated using multivariable binary logistic regression, with overweight as reference due to the nonlinear relationship. Both normal-weight (odds-ratio [OR] 2.32, 95% confidence interval [CI] 1.30–4.14) and obese (OR 2.25, 95% CI 1.08–4.71) patients had an increased risk of poor functional outcome, driven by deaths only in the normal-weight. In normal-weight, s-IGFBP-1 modestly attenuated (8.3%) this association. In the obese, the association was instead attenuated by HOMA-IR (22.4%) and hs-CRP (10.4%). Thus, a nonlinear relation between BMI and poor 7-year functional outcome was differently attenuated in the normal-weight and the obese.

## 1. Introduction

Ischemic stroke (henceforth stroke) has high mortality and disability worldwide [1]. Among stroke survivors, the recovery period sometimes extends for years [2]. High body mass index (BMI) is a risk factor for developing stroke, with increased risks for the overweight (BMI 25–30) and obese (BMI > 30), as compared with the normal-weight (BMI 18.5–25) individuals [3]. In contrast, in the poststroke period, it has been puzzling why normal-weight patients with better overall risk profiles do not also have the best recovery and survival [4,5]. In fact, BMI above the normal range has been associated with better prognosis in both stroke and a broad range of cardiovascular diseases (CVD), which is often referred to as the “obesity paradox” [5,6,7,8]. Most often, the overweight range of BMI 25–30 has been associated with better outcomes, especially mortality, than normal range BMI (18.5–25) or obese BMI (>30) [6,8]. This holds, although BMI has been criticized for not being a perfect measure of overweight. For example, ethnicity, sex, visceral fat distribution, and muscle mass may be important confounding factors affecting true metabolic burden [9]. However, despite these limitations, BMI has been shown as one of the better indices to predict dyslipidemia [10].

There are conflicting explanations for the obesity paradox. One hypothesis is that the obesity paradox is merely a result of BMI measurements in prevalent CVD, with disease-related weight loss and survival bias [11]. However, other studies have found an obesity paradox not only for prevalent stroke patients with BMI measurements in the poststroke phase, but also for incident stroke with BMI measurements in a healthy state before the event [8]. Consequently, one proposed explanation for the obesity paradox is an increased metabolic reserve in adipose tissue and skeletal muscle, which protects against the increased catabolic drive and suppressed anabolic stimulation after stroke [12]. It has also been proposed that possible mechanisms for the obesity paradox could be specific anti-inflammatory effects of adipose tissue [13], as well as different mechanisms of hypertension between normal-weight (BMI 18.5–25) and overweight (BMI 25–30) patients [14].

Another part of the obesity paradox is insulin resistance (IR), which is related to BMI and linked to an increased risk of stroke and other CVD [15]. IR may be quantified by several methods, including the homeostasis model assessment of insulin resistance (HOMA-IR). HOMA-IR is easier to monitor than the golden standard euglycemic clamp method, and there is a high correlation between IR estimations using HOMA-IR and the euglycemic clamp method [16]. Impaired HOMA-IR is a well-known risk factor for incident stroke [17], as well as for poor poststroke functional outcome and mortality [18]. Insulin resistance, in turn, is also related to general inflammation, as indexed by, for example, serum levels of C-reactive protein (CRP, for review see: [19]). Although CRP is produced at higher levels in the liver in response to infections and high-degree inflammation [19], there is also a low-grade inflammation with CRP released from the vascular endothelium thought to mirror atherosclerosis [20]. Regardless of the exact mechanism of CRP elevation, it associates with general stroke [21] and CVD risk [22]. After a stroke, CRP has also been shown to independently associate with poor functional outcome [23].

Related to IR is insulin-like growth factor-binding protein-1 (IGFBP-1), which typically binds insulin-like growth factor 1 (IGF-1), resulting in reduced IGF-1-activity [24,25]. Levels of s-IGFBP-1 are negatively correlated with IR [26,27] and BMI [28]. Furthermore, several studies have shown that high levels of s-IGFBP-1 are associated with worse outcome after cardiovascular events [25,29,30]. In line with this, high s-IGFBP-1 is considered a marker for general catabolic states and borderline cachexia [31]. However, to our knowledge, no study has investigated whether s-IGFBP-1 could explain the obesity paradox in stroke.

Here, we aimed to examine the obesity paradox concerning long-term poststroke outcomes and possible mediators. We have also included HOMA-IR, a common cardiovascular risk factor in the analysis, and high-sensitivity C-reactive protein (hs-CRP) as a marker of chronic inflammation [19]. Therefore, we investigated ischemic stroke patients from the Sahlgrenska Academy Study on Ischemic Stroke (SAHLSIS) cohort, regarding relationships between BMI, s-IGFBP-1, HOMA-IR, and functional outcome evaluated by modified Ranking scale (mRS) at poststroke follow-up at 7 years. For regressions, postulating from earlier studies of the obesity paradox that there is a nonlinear relationship between BMI and outcome [5,6,7,8], we used overweight (BMI 25–30) as a reference versus the other BMI categories, i.e., for the convenience of understanding adjustments.

## 2. Results

### 2.1. Baseline Characteristics and Correlations

The baseline characteristics of the included patients (n = 451) are summarized in Table 1. As the aim was to investigate the proposed obesity paradox, data in Table 1 are also shown for three BMI categories (normal-weight, overweight, and obesity). Age and sex distributions differed slightly between BMI categories, where the obese were 3.5 years older than the normal-weight, and the normal-weight group comprised more females than the overweight group. There were also higher proportions of hypertension and diabetes in patients with higher BMI category. In contrast, smoking was more frequent in the normal-weight category, as expected. In terms of s-IGFBP-1, it was lower in higher BMI categories. Specifically, the mean s-IGFBP1 in obese (BMI > 30) patients was 52% of that in normal-weight (BMI 18.5–25) patients. Hs-CRP was numerically higher in the normal-weight (BMI 18.5–25) and obese (BMI > 30) patients. National Institutes of Health Stroke Scale (NIHSS) score and frequency of previous stroke did not significantly differ between the BMI categories.

Correlation matrices for the factors of investigation are given in Table 2. The Spearman correlation analyses showed significant correlations between BMI, s-IGFBP-1, HOMA-IR, and hs-CRP. Of note, s-IGFBP-1 displayed a small significant negative correlation with BMI (r = −0.24) and a moderate negative correlation with HOMA-IR (r = −0.32). Additionally, HOMA-IR showed a moderate positive correlation with BMI (r = 0.38) and a small positive correlation with hs-CRP (r = 0.22).

### 2.2. BMI and Poststroke Functional Outcome

The number of patients with poor poststroke functional outcomes at 3 months, 2 years, and 7 years in normal-weight, overweight, and obesity is presented in Table 3. The number of deaths and previous strokes, used for the sensitivity analysis below, are shown in Supplementary Table S2. At 3 months and 2 years poststroke, the proportion of cases with poor functional outcomes was similar in the three BMI categories. At 7 years of follow-up, there was a larger proportion of patients with poor functional outcome, with significant differences between the three BMI categories. Specifically, at 7 years poststroke, only 28.0% of the overweight (BMI 25–30) patients had poor functional outcome compared to 42.2% in the normal-weight (BMI 18.5–25) patients (*p* = 0.004) and 48.7% in the obese (BMI > 30) patients (*p* = 0.001).

**Figure 1:**
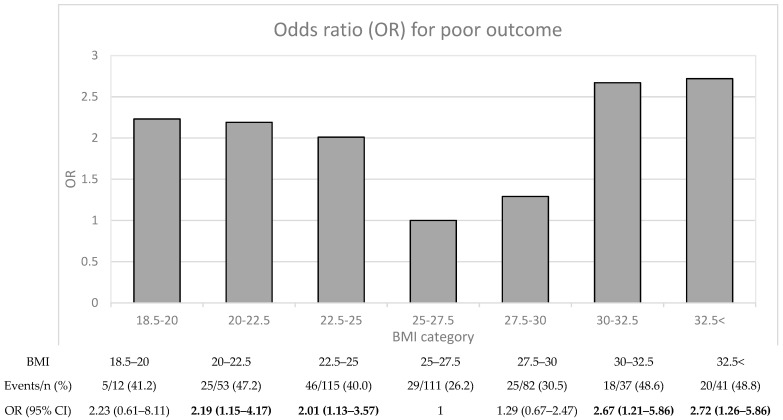
Poor functional outcome after 7 years poststroke. Odds ratios (ORs) for poor functional outcome (mRS 3–6) with 95% CI were calculated using binary logistic regression. ORs are adjusted for age and sex (Model 1). ORs with statistically significant *p*-values (<0.05) are shown in bold. Abbreviations: BMI, body mass index; mRS, Modified Rankin Scale.

To investigate the association between BMI and poststroke functional outcome in more detail, we divided the patients into seven BMI subcategories (Figure 1), and performed binary regressions adjusted for sex and age (Model 1). Overall, the nonlinear relationship between BMI and 7-year functional outcome was retained. Specifically, with the BMI group of 25.0–27.5 as a reference, binary regression showed the lowest relative ORs for the risk of poor 7-year functional outcome in the overweight BMI subcategories (BMI 25–27.5 and 27.5–30). The ORs for the risk of poor 7-year functional outcome ranged from 2.01 to 2.72 for normal-weight (BMI 18.5–25) and obese (BMI > 30) BMI subcategories.

As functional outcomes were significantly different by BMI category only for the 7-year follow-up, we investigated the reverse, namely, whether the baseline parameters differed in patients with good or poor functional outcome after 7 years (Table 4). The patients with poor 7-year functional outcome were older and had a larger proportion of severe strokes, diabetes, previous strokes, and sedentary lifestyle compared to the patients with favorable outcome, and they also displayed higher levels of s-IGFBP-1, HOMA-IR, and hs-CRP. In contrast, there were no baseline differences in sex, BMI, hypertension, blood pressure, smoking, or LDL levels. The absence of a difference in baseline BMI levels comparing good and poor outcome may be surprising, but is expected given the shown nonlinear relationship in Figure 1.

### 2.3. The Relation between BMI, HOMA-IR, and s-IGFBP-1 and Poststroke Functional Outcome

Having observed a nonlinear association between BMI and functional outcome (Figure 1), we performed the binary logistic regressions in different strata of BMI, as there may potentially exist different relations between BMI, HOMA-IR, and s-IGFBP-1 in the lower or higher ranges of BMI (Table 5a,b). Adjustments were made for sex and age (Model 1), cardiovascular risk factors (Model 2), and additionally for either BMI-category, s-IGFBP-1, HOMA-IR, diabetes, or hs-CRP.

In Table 5a, we show the associations with poor 7-year functional outcome in the subpopulation with BMI 18.5–30 (normal-weight and overweight) for three parameters: normal-weight (BMI 18.5–25) vs. overweight (BMI 25–30), increases in Log10-s-IGFBP-1, and increases in Log10-HOMA-IR. For these regressions, Model 2 adjustments (cardiovascular risk factors except diabetes) should be regarded as the “crude” model for comparison. As expected, there was a significantly higher risk of poor functional outcome after 7 years in normal-weight patients than in overweight patients (OR 2.32, 95% CI 1.30–4.14). Adjusting for s-IGFBP-1 moderately attenuated this association by 8.3% (OR 2.21, 95% CI 1.21–4.02). In comparison, neither hs-CRP (Model 2 + hs-CRP) nor HOMA-IR (Model 2 + HOMA-IR) attenuated the association. Moreover, in the middle and right panels of Table 5a, we present the associations between increases in Log10-s-IGFBP-1 or Log10-HOMA-IR and the risk of poor 7-year functional outcome among the patients with BMI 18.5–30 (normal-weight and overweight), with corresponding adjustments. Interestingly, increases in Log10-s-IGFBP-1 retained statistical significance in all models (Table 5a, middle panel), whereas the association with Log10-HOMA-IR was attenuated below the significance level by adjustment for hs-CRP or diabetes (Table 5a, right panel).

In Table 5b, we show the associations with poor 7-year functional outcome in the subpopulation with BMI > 25 (overweight and obese) for the following three parameters: overweight (BMI 25–30) vs. obese (BMI > 30), increases in log10-s-IGFBP-1, and increases in log10-HOMA-IR. Being obese (BMI > 30) was associated with an increased risk of poor 7-year functional outcome, but this association was attenuated below the significance level by adjustment for hs-CRP and HOMA-IR. HOMA-IR and hs-CRP showed attenuation percentages of 22.4%, and 10.4%, respectively. In contrast, adjustment for IGFBP-1 accentuated the association (Table 5b, left panel). Concerning increases of Log10-s-IGFBP-1 in the subpopulation with BMI > 25 (overweight and obese), the unadjusted OR was not significant (Table 5b, middle panel), which limits the interpretation of the following adjustments, although an accentuation was found in Model 2. In contrast, increases of Log10-s-HOMA-IR in the subpopulation with BMI > 25 had a significant unadjusted OR of 5.34. However, this OR was attenuated to just below significance level by Model 2 adjustment, but was accentuated by adjustment for s-IGFBP-1 and hs-CRP (Table 5b, right panel).

To investigate if deaths (mRS 6) or previous strokes at baseline were driving the associations for BMI and poor functional outcome (mRS 3–6), we performed a sensitivity analysis in the logistic regressions by adding death, previous strokes, and sedentary lifestyle as covariates (Supplementary Tables S1 and S2). First, deaths are shown specifically for the different time points and BMI categories, in analogy to Table 3. Supplementary Table S1 shows the overall percentage of deaths in each BMI category as well as deaths from ischemic stroke, hemorrhagic stroke, cardiovascular cause, and other causes. Essentially, the pattern with fewer deaths in the overweight group was preserved. Specifically, in patients with poor outcome (n = 168), after 7 years there were 47% deaths (mRS 6) and 53% with functional disability (mRS 3–5). In the sensitivity analysis using binary logistic regression by adding deaths as a covariate (Supplementary Table S2), death attenuated the increased risk of 7-year poor outcome in normal-weight patients by 82.6%, and thus explained a major part of the poor functional outcomes. However, for the obese, the attenuation by death was negligible (1.6%). Thus, the relations appear to be largely driven by 7-year poststroke deaths in the normal-weight group, but not in the obese group. A history of previous stroke did not attenuate the increased risk of poor outcome in any of the BMI groups. Sedentary lifestyle attenuated poor functional outcome in the obese, but not in the normal-weight group (Supplementary Table S2), similar to the pattern of HOMA-IR (Table 5).

### 2.4. Poor Functional Outcome in IGFBP-1, HOMA-IR, and BMI Categories

In Table 6 and Table 7, we analyzed the three BMI categories regarding poor functional outcome according to medians of s-IGFBP-1 (Table 6) and HOMA-IR (Table 7). The patients with complete data on these three parameters at 7 years poststroke were fewer (n = 330), but nevertheless had similar baseline parameters (Supplementary Table S3). The reference category in Table 6 was overweight (BMI 25–30) patients with s-IGFBP-1 below the median, and in Table 7, the reference category was overweight (BMI 25–30) patients with HOMA-IR below the median. Numbers and unadjusted ORs of patients with poor functional 7-year outcomes are given.

In Table 6, these analyses showed that the risk of poor 7-year functional outcome was increased in the normal-weight (BMI 18.5–25) and obesity (BMI > 30) categories as compared to the overweight (BMI 25–30) category. Furthermore, the risk of poor functional outcome was more pronounced for patients with high IGFBP-1 in all three BMI categories (normal-weight: OR 4.41, 95% CI 2.00–9.73; overweight: OR 2.5, 95% CI 1.14–5.51; obesity: OR 4.67, 95% CI 1.79–12.1) compared with the reference group of overweight patients with low s-IGFBP-1.

A similar pattern was found for HOMA-IR (Table 7). Normal-weight (BMI 18.5–25) and obesity (BMI > 30) were both associated with an increased risk of poor functional 7-year outcome regardless of whether HOMA-IR was low or high as compared to the reference group (overweight (BMI 25–30) with low HOMA-IR). For normal-weight patients with high HOMA-IR, the OR for poor prognosis was more pronounced (OR 4.41, 95% CI 2.00–9.73). Obese patients with high HOMA-IR showed the worst prognosis (OR 6.15, 95% CI 2.36–16.1), which was a considerably higher OR than that of obese patients with low HOMA-IR (OR 2.63 95%, CI 0.98–7.05).

## 3. Discussion

### 3.1. Nonlinear Associations between BMI and Poor Functional Outcome—Relations with IGFBP-1 and Insulin Resistance

In line with earlier results [5,32], we found an obesity paradox in the sense that normal-weight (BMI 18.5–25) patients did not have the most favorable 7-year functional outcome, despite their overall better cardiovascular risk factor profile regarding hypertension, diabetes, BMI, LDL, and HOMA-IR levels (Table 1). Nevertheless, smoking was more abundant, and hs-CRP was nonsignificantly higher, both of which were adjusted for in Model 2. The lowest risk of poor functional outcome was in the overweight (BMI 25–30) patients, whereas normal-weight (BMI 18.5–25) and obese (BMI > 30) patients had significantly worse 7-year functional outcome. Our results therefore support an obesity paradox in terms of long-term functional outcome, resembling a U-shaped nonlinear relation, as also recently reported on 3-month poststroke functional outcomes [5]. The obesity paradox should perhaps even be called a normal-weight paradox, as we discuss further below. Furthermore, we investigated whether the association between normal-weight (BMI 18.5–25) and the obese (BMI > 30) and the risk of poor functional outcome was dependent on HOMA-IR and s-IGFBP-1. In line with our hypothesis, s-IGFBP-1 slightly attenuated (8.3%) the association for poor functional outcome in the normal-weight (BMI 18.5–25) compared to overweight (BMI 25–30), but not to the degree that it could be regarded as a robust mediator. In contrast, the factors that attenuated the association for poor prognosis in the obese (BMI > 30) compared to the overweight (BMI 25–30) were HOMA-IR (22.4%) and hs-CRP (10.4%). Furthermore, adjustments for HOMA-IR showed opposite attenuations as compared to those of s-IGFBP-1 for both normal-weight vs. overweight and obese vs. overweight. Thus, although BMI, s-IGFBP-1, and HOMA-IR are moderately intercorrelated, they only mediate each other’s effects on poststroke functional outcome to a small extent. The associations for poor functional outcome were largely driven by deaths in the normal-weight but not in the obese category.

### 3.2. Different Attenuation for Poor Functional Outcome in the Normal-Weight and the Obese

Considering the nonlinear relation between BMI and poststroke functional outcome, there could potentially be different confounding or mediating effects in the different ranges of BMI. The findings that high s-IGFBP-1 is associated with poor functional outcome (Table 4), and that high s-IGFBP-1 is found in patients with lower BMI (Table 1), could suggest that IGFBP-1 is mediating the poor functional outcome for the normal-weight. However, the attenuation by s-IGFBP1 was modest (8.3%) when introduced as a covariate. Nevertheless, s-IGFBP-1 was the only examined factor that attenuated the OR in the normal-weight vs. overweight BMI comparison. In contrast, another investigated factor, hs-CRP, which could have explained part of this relationship, did not exhibit any attenuation. The effects of adjustments for covariates were different in the overweight and obese subpopulations with BMI > 25 (Table 5b, left panel). Specifically, the relationship between poor functional outcome in the obese compared to the overweight was attenuated by HOMA-IR (22.4%) and hs-CRP (10.4%), which thus could partly explain the associations in the obese spectrum.

### 3.3. Impact and Additive Effects of IGFBP-1 and HOMA-IR in the Different BMI Categories

In Table 5a,b in the middle panels, we demonstrate that s-IGFBP-1 is indeed a determinant of poststroke functional outcome in the subpopulation with BMI 18.5–30 (normal-weight to overweight), with more robust ORs than that observed for HOMA-IR. However, in the subpopulation with BMI > 25 (overweight to obese), s-IGFBP-1 may be a weaker risk factor than HOMA-IR in the unadjusted and Model 1 associations. However, it is difficult to be certain that HOMA-IR is more important than s-IGFBP-1, as there are also some accentuations in the further adjustments.

Moreover, adding s-IGFBP-1 as a covariate did not attenuate the association between increases in HOMA-IR and poor poststroke functional outcome in BMI 18.5–30. Altogether, this could suggest that s-IGFBP-1 is not a mediator of the detrimental effects of high HOMA-IR, but to some degree, a mediator (or confounder) for better poststroke functional outcomes in overweight (BMI 25–30) patients.

Overall, Table 6 and Table 7 imply that patients with overweight (BMI 25–30) and low s-IGFBP-1 or low HOMA-IR demonstrated the largest proportion of good 7-year functional outcome. It is noteworthy that also in the overweight (BMI 25–30) patients with high s-IGFBP-1 (Table 6), and high HOMA-IR (Table 7), there are statistically significant higher risks for poor 7-year functional outcomes. Other studies have shown that normal-weight (BMI 18.5–25) patients with high HOMA-IR, the so-called Metabolically Unhealthy Normal Weight (MUHNW), have worse poststroke functional outcomes and increased mortality [33]. This is supported by data from Table 7, but there is also a significant risk increase for the normal-weight with low HOMA-IR compared to both categories of overweight patients. Finally, Table 6 and Table 7 shows that both high s-IGFBP-1 and HOMA-IR are rather independent risk factors in all the weight categories.

### 3.4. Effect of Follow-Up Time

Additionally, the association between BMI at index stroke and functional outcome was more marked when the observation times were longer. Although the association patterns were numerically in the same direction for the 2-year follow-up, there was a distinctly higher difference between the BMI categories in the risk of poor functional outcome with longer follow-up time (a significant difference was observed only at the 7-year follow-up). More pronounced associations with poststroke outcomes during longer follow-up have previously been observed for HOMA-IR [34] and s-IGFBP-1 [25], which could indicate that the factors are related. This could also indicate that these factors are less related to early recovery processes in the poststroke phase and more to long-term effects of metabolic factors, priming long-term recovery and deaths. The accentuation by long follow-up could also support that overweight BMI is a factor of resilience for deterioration of functional outcomes. However, our data only allowed analysis of poststroke cardiovascular risk factors that were known at index (cardiovascular adjustments in Model 2). Even so, the different Model 2 adjustments showed relatively small attenuations. Thus, it appears that the augmentation over time of the effects of BMI, HOMA-IR, and s-IGFBP-1 on functional outcome appear to be relatively independent, despite the moderate intercorrelation between these factors. It should also be acknowledged that the trajectories of BMI changes in the different baseline BMI categories would have aided interpretation. In fact, changes in BMI, e.g., weight reductions, would probably be a powerful predictor of a poor functional outcome as indicated by the results of previous studies [35,36]. In this aspect, weight loss is most often resulting from severe strokes with loss of appetite, decreased feeding ability, and muscle mass reduction. However, follow-up BMI was not collected in our study, which is a limitation.

Could the time-dependent association between BMI and poor functional outcome (mRS 3–6) be driven by the proportion of deaths (mRS 6)? Interestingly, in the sensitivity analysis, we observed that death in a high degree attenuated the increased risk of poor functional outcome for the normal-weight, but not for the obese. This indicates that death indeed is largely driving the associations between BMI and 7-year poor functional outcomes in the normal-weight but not in the obese.

### 3.5. Strengths and Limitations

Our study had a relatively large sample size regarding s-IGFBP1 measurements and poststroke functional outcome. In the multivariate regression models, there was a sufficient number of patients and events to adjust for relevant potential confounders, as argued by the algorithms supplied by Peduzzi and coworkers [37]. Moreover, we only included young and middle-aged predominantly white patients, as the maximum age for inclusion in SAHLSIS was 70 years. This could be important to consider when comparing our results with similar studies from other ethnic groups in Asia [38,39] or cohorts with older patients. Additionally, our study included diabetic patients, even though insulin treatment can interfere with HOMA-IR measurements [34]. However, this is mainly a problem when HOMA-IR is not analyzed under steady-state conditions [40], which it was in our study (morning fasting hours). Still, there is a limitation of the HOMA-IR values for diabetic patients as we lack records of medication, and because acute ischemic stroke, per definition, is a state of non-steady-state. However, since HOMA-IR was not the main focus of this study, we found it pertinent to include diabetic patients to provide a better picture of the role of BMI. Furthermore, the day of blood sampling was not standardized, and ranged from day 1 to 10 after index stroke (median 4 days). Therefore, some patients could theoretically be in a state of stress hyperglycemia. However, hs-CRP, which reflects the general stress response, did not statistically significantly affect the associations, except for in the obese subpopulation (Table 5). HbA1c would have been a suitable variable to include, but was not included in the original protocol of the study, which is a limitation. Furthermore, we do not have records of the participants’ amount of rehabilitation or changes in medications during the follow-up, which is also a limitation.

### 3.6. The Obesity Paradox and Other Possible Mechanisms

Although an obesity paradox has been reported for poststroke prognosis [6,8], the magnitude and relations to other factors have varied. One study found no obesity paradox in patients with HOMA-IR below the median, whereas normal-weight patients with high HOMA-IR had a worse prognosis after ischemic stroke [33]. We could not replicate these findings, as we found that normal-weight (BMI 18.5–25) patients, regardless of high or low HOMA-IR, had a worse prognosis than overweight (BMI 25–30) patients with high HOMA-IR. However, the mentioned study [33] had a follow-up time of only one year compared to the present study with 7 years of follow-up. Furthermore, the previous study had different BMI criteria according to the standards for Asian participants [39], which could explain some of the different results.

Some serum factors could be of importance for the obesity paradox that we did not analyze. One study found that normal-weight (BMI 18.5–25) participants with hypertension secrete more catecholamines and renin during exercise than obese (BMI > 30) participants [14]. If extrapolated to our long-term study, this could contribute to the unfavorable prognosis in normal-weight individuals. Additionally, TNF-α, an inflammatory cytokine involved in numerous pathological mechanisms, and soluble TNF-α receptors, might be important in explaining the obesity paradox [13,41]. Indeed, TNF-α is released in the brain during ischemic stroke and has been shown to mediate cell death [41]. Furthermore, TNF-α inhibition has been shown to promote neurological recovery in rodent models [42,43]. In vivo, experiments have shown that soluble TNF-α receptors in the circulation, released from adipose tissue, can bind TNF-α [13]. A larger adiposity could therefore neutralize the negative effects of local TNF-α synthesis after stroke. Accordingly, one theory explaining an obesity paradox in the poststroke recovery phase is that inflammatory TNF-α released during ischemic events like stroke may be bound mainly by circulating TNF-α receptors in individuals with more subcutaneous adipose tissue, leading to less inflammatory TNF-α in overweight and obese individuals [44].

## 4. Materials and Methods

### 4.1. Study Population

The present study included ischemic stroke cases from the hospital-based prospective observational, longitudinal cohort study SAHLSIS, described previously [45,46] and in the online supplement. Briefly, adult patients aged <70 years with first-ever or recurrent acute ischemic stroke were recruited consecutively at four Stroke Units in western Sweden during 1998–2003 (n = 600). There were 458 patients who had both measurements of BMI and either HOMA-IR or s-IGFBP-1 at index stroke. Out of these, n = 7 had a BMI below normal-weight (<18.5). Since adult underweight below 18.5 is uncommon and often is associated with cachexia [47], these individuals were excluded, rendering the 451 study participants (Supplementary Figure S1, flow chart, Supplementary Materials). BMI values were grouped according to the classification defined by the World Health Organization (WHO): adult normal-weight as a BMI 18.5–25 kg/m^2^, overweight as a BMI 25–30 kg/m^2^, and obesity as a BMI above 30 kg/m^2^, which is suitable for a western population [48]. BMI, hypertension, diabetes, sedentary lifestyle, and smoking were defined as described previously [45,46,49], and are also presented in the Supplementary Materials. Functional independence was evaluated by mRS at poststroke follow-up at 7 years. Of these, there were also n = 432 with mRS at 3 months and n = 449 with mRS at 2 years poststroke. For the 7-year follow-up, there were n = 330 with complete data for both s-IGFBP1 and HOMA-IR. Written informed consent was obtained from all study participants or next of kin. SAHLSIS and the follow-up studies were approved by the Regional Ethics Review Board in Gothenburg, Sweden (#Ö469-99 and #413-04, #T665-07, T586-13).

### 4.2. Stroke Severity and Functional Outcome

In SAHLSIS, the maximum stroke severity within the first 7 days of admission to the hospital was assessed by the Scandinavian Stroke Scale (SSS), and converted to the more frequently used NIHSS using the established algorithm NIHSS = 25.68 − 0.43 × SSS [50]. Of note, recruitment to *SAHLSIS* took place before recanalization therapy was part of clinical routine treatment. Functional outcome at 3 months, 2 years, and 7 years after index stroke was assessed using the mRS scale. Over the years, these were performed by one neurology specialist physician at baseline and at the 3-month follow-up. At the 2-year and 7-year follow-ups, a research nurse, trained in stroke medicine by the research specialist physician, assessed functional outcome. Stroke severity was assessed at the inpatient clinic, while all other functional assessments including mRS were performed outpatient on follow-up occasions outside the hospital ward.

### 4.3. Blood Sampling and Protein Measurement

Venous blood samples were collected in the acute phase, within a range of 1–10 days (median 4 days) after the index stroke. Blood sampling was performed in the morning hours, after an overnight fast of >8 h, where serum was isolated within 2 h by centrifugation at 2000× *g* at 4 °C for 20 min, aliquoted, and stored at −80 ◦C pending analyses. S-IGFBP-1 was analyzed by an enzyme-linked immunosorbent assay (ELISA) using a commercial kit from Mediagnost, Reutlingen, Germany. The inter-assay coefficient of variation was 14.6%, and the intra-assay coefficient of variation was 4.5%, as reported [25]. Due to the relatively high inter-assay variation for s-IGFBP-1, an inter-assay correction factor was used by placing three original serum samples in each 96-well plate. All blood and plasma concentrations of insulin and glucose were analyzed using standardized methods at the Department of Clinical Chemistry at the Sahlgrenska University Hospital. Calculation of HOMA-IR was done with an algorithm: fasting insulin (microU/L) × fasting glucose (nmol/L)/22.5 [16]. High-sensitivity CRP (hs-CRP) was analyzed by a solid-phase chemiluminescent immunometric assay on Immulite 2000 (Diagnostic Products Corp, Los Angeles, CA, USA) with reagents as directed by the manufacturer.

### 4.4. Statistical Analysis

The statistical analyses were performed using the IBM^®^ SPSS^®^ ver. 25 software (SPSS Inc., Armonk, NY, USA). Continuous data are presented as mean values and 95% confidence intervals (CIs). The Shapiro–Wilk test was performed to test normality, with no variables showing normal distribution. Due to skewed distribution, the exposure variables s-IGFBP-1, hs-CRP, and HOMA-IR were log-transformed in the statistical analyses.

For descriptive purposes, Mann–Whitney U-tests were performed to examine between-group differences in continuous variables, and Chi square tests to examine differences in categorical values. Correlation analysis was performed according to Spearman, and correlation strengths (rho values; r) were reported according to Cohen [51]. Thus, we report the magnitude of r as very small for r < 0.1, small for r < 0.1 to 0.3, moderate for r < 0.3 to 0.5, and large for r > 0.5. Multivariable binary logistic regression was used to determine odds ratios (ORs) for the associations with poor functional outcome.

Adjustments were made for sex, age (Model 1), and additionally for conventional vascular risk factors (hypertension, smoking, and LDL), and acute stroke severity (Model 2). In the adjustments of the regression analysis, attenuation percentages were calculated as ([Unadjusted OR-1] − [Adjusted OR-1])/(Unadjusted OR-1). For further details on attenuation percentage, see the Supplementary Materials. The appropriate number of covariates was based on the included participants and the fraction of outcomes as reported by Peduzzi and coworkers [37]. For LDL, which had the most missing values (N = 63 or 14.0%, Table 1), imputation was used to replace the missing values with the mean LDL value [49]. Statistical significance was defined as a two-tailed *p* < 0.05.

## 5. Conclusions

We conclude that serum IGFBP-1 is an important prognostic factor for long-term poststroke functional outcome, and might play a minor part in what is commonly referred to as the “obesity paradox”. However, considering our results from the binary logistic regression analyses, it may at most partially explain why normal-weight (BMI 18.5–25) patients have an increased risk of poor outcome compared to overweight (BMI 25–30) patients. In contrast, in the poor outcome of obese (BMI > 30) patients, there was a somewhat higher degree of attenuation by HOMA-IR and hs-CRP.

Furthermore, our results support the notion that the phenomenon called “the obesity paradox” could instead be regarded as “the normal-weight paradox” [4], as indicated by our findings of a nonlinear association with the best poststroke functional outcome in the overweight (BMI 25–30) group, and not in the normal-weight (BMI 18.5–25) group as expected. For further studies, the relationships between BMI, TNF-α, soluble TNF-α receptors, catecholamines, renin, and IGFBP-1 need more in-depth exploration. A further understanding of the exact mechanisms underlying the obesity paradox might also suggest new and tailored pharmacological therapies for stroke recovery.

## Figures and Tables

**Table 1:** Associations and differences between normal-weight, overweight, and obese stroke patients in baseline values and outcome.

	All	A. Normal-Weight	*p*	B. Overweight	*p*	C. Obese	*p*
Variable	n	(BMI 18.5–25)	A vs. B	(BMI 25–30)	B vs. C	(BMI > 30)	A vs. C
All patients (N, %)	451	180 (100)	NA	193 (100)	NA	78 (100)	NA
Females (N, %)	163	74 (41.1)	**0.034**	59 (30.6)	0.212	30 (38.5)	0.692
Males (N, %)	288	106 (58.9)	**0.034**	134 (69.4)	0.212	48 (61.5)	0.692
Age, years (95% CI)	451	54.9 (53.2–56.6)	**0.042**	57.9 (56.6–59.2)	0.54	58.4 (56.4–60.4)	**0.048**
BMI, kg/m^2^ (95% CI)	451	22.8 (22.6–23.1)	**<0.001**	27.3 (27.1–27.5)	**<0.001**	33.7 (32.9–34.5)	**<0.001**
Hypertension (N, %)	451	85 (47.2)	**0.001**	123 (63.7)	**0.021**	61 (78.2)	**<0.001**
Systolic BP, mmHg, mean (95% CI)	442	143 (138–147)	**0.010**	148 (145–152)	0.586	150 (145–156)	**0.011**
Diastolic BP, mmHg, mean (95% CI)	441	82 (80–85)	**0.039**	85 (83–87)	0.696	86 (83–88)	**0.041**
Smoking (N, %)	451	77 (42.8)	0.166	69 (35.8)	0.326	23 (29.5)	**0.044**
Diabetes (N, %)	451	19 (10.6)	**0.003**	42 (21.8)	0.119	24 (30.8)	**<0.001**
LDL, mmol/L, mean (95% CI)	388	3.17 (3.03–3.31)	**0.008**	3.47 (3.32–3.62)	0.632	3.40 (3.12–3.69)	0.173
Imputed LDL, mmol/L, mean (95% CI)	451	3.18 (3.06–3.31)	**0.007**	3.45 (3.32–3.58)	0.643	3.39 (3.16–3.62)	0.123
hs-CRP, mg/L, mean (95% CI) #	431	11.0 (7.39–14.6)	0.551	7.78 (5.63–9.93)	**0.009**	10.7 (6.95–14.4)	**0.006**
Sedentary lifestyle (N/tot N, %)	424	22 (12.9)	0.668	26 (14.4)	**<0.001**	25 (34.2)	**<0.001**
Previous stroke (N/tot N, %)	451	35 (19.4)	0.953	38 (19.6)	0.932	15 (19.2)	0.968
NIHSS, mean (95% CI)	451	5.20 (4.35–6.04)	0.858	4.49 (3.81–5.18)	0.043	5.35 (4.27–6.44)	0.096
s-IGFBP1, µg/L, mean (95% CI)	341	8.70 (7.14–10.3)	**0.017**	7.07 (5.65–8.48)	**0.011**	4.50 (3.49–5.51)	**<0.001**
Insulin, microU/L, mean (95% CI)	430	12.8 (10.6–14.9)	**<0.001**	14.7 (13.0–16.4)	**<0.001**	23.6 (19.0–28.0)	**<0.001**
Glucose, nmol/L, mean (95% CI)	427	5.92 (5.56–6.27)	**<0.001**	6.64 (6.26–7.01)	0.309	6.75 (6.17–7.33)	**<0.001**
HOMA-IR, mean (95% CI)	413	3.55 (2.86–4.24)	**<0.001**	4.69 (3.90–5.48)	**<0.001**	6.65 (5.43–7.88)	**<0.001**

Values are presented as means and 95% CI or percentage fraction. The *p*-values are based on Mann–Whitney U analysis (all continuous variables were non-normally distributed) and by Chi square analysis (categorical variables: sex, hypertension, smoking, diabetes). Statistically significant *p*-values (<0.05) are shown in bold. # Due to the non-normal distribution of hs-CRP, the Mann–Whitney U detects a significant difference between normal-weight and obese (B. vs. C.), but not for normal-weight vs. overweight (A. vs. B.). Abbreviations: BMI, body mass index; BP, blood pressure; hs-CRP, high-sensitivity C-reactive protein; HOMA-IR, homeostatic model assessment of insulin resistance; LDL, low-density lipoprotein; s-IGFBP1, serum levels of insulin-like growth factor-binding protein-1; NIHSS, National Institutes of Health Stroke Scale; NA, not applicable.

**Table 2:** Correlations between BMI, HOMA-IR, s-IGFBP-1, and hs-CRP.

Parameter	BMI	s-IGFBP-1	HOMA-IR	hs-CRP
BMI, r (p)	NA	**−0.24 (<0.001)**	**0.38 (<0.001)**	0.07 (0.208)
s-IGFBP1, r (p)	**−0.24 (<0.001)**	NA	**−0.32 (<0.001)**	0.05 (0.370)
HOMA-IR, r (p)	**0.38 (<0.001)**	**−0.32 (<0.001)**	NA	**0.22 (<0.001)**
hs-CRP, r (p)	0.07 (0.21)	0.05 (0.370)	**0.22 (<0.001)**	NA

N = 330. Values are presented as correlation coefficient (r) and corresponding p-value, derived from Spearman calculations. Correlations with statistically significant *p*-values (<0.05) are shown in bold. Abbreviations: BMI, body mass index; hs-CRP, high-sensitivity C-reactive protein; HOMA-IR, homeostatic model assessment of insulin resistance; s-IGFBP1, serum levels of insulin-like growth factor-binding protein-1; NA, not applicable.

**Table 3:** Poor functional outcome after 3 months, 2 years, and 7 years poststroke.

Poor Outcome—Time Point	n	*p* (3-Group)	A. Normal-Weight (BMI 18.5–25)	*p* A vs. B	B. Overweight (BMI 25–30)	*p* B vs. C	C. Obese (BMI > 30)	*p* A vs. C
3 months [n/total n in category, (%)]	432	0.956	35/172 (20.3)	0.795	36/187 (19.2)	0.814	15/73 (20.5)	0.972
2 years [n/total n in category, (%)]	449	0.508	39/180 (21.7)	0.287	33/191 (17.2)	0.389	17/61 (27.9)	0.982
7 years [n/total n in category, (%)]	451	**0.001**	76/180 (42.2)	**0.004**	54/193 (28.0)	**0.001**	38/78 (48.7)	0.337

Numbers (n) of patients are shown for poor outcome (mRS 3–6), total n, and percentage fraction. The *p*-values are derived from Chi square analysis, the first column showing all three groups compared, and in the following columns, specific comparisons, as indicated. Statistically significant *p*-values (<0.05) are shown in bold. Abbreviations: BMI; body mass index, mRS; Modified Rankin Scale.

**Table 4:** Baseline characteristics of the patients with good and poor functional outcomes (mRS 3–6) after 7 years poststroke.

Variable	N	Entire Cohort	Good Outcome (mRS 0–2)	Poor Outcome (mRS 3–6)	Good vs. Poor, *p*-Value
All patients, N (%)	451	451 (100)	283 (100)	168 (100)	NA
Females, N (%)	163	163 (36.1)	104 (36.7)	59 (35.1)	0.728
Males, N (%)	288	288 (63.8)	179 (63.3)	109 (64.9)	0.728
Age, years (95% CI)	451	56.8 (55.8–57.7)	55.1 (53.9–56.3)	59.7 (58.2–61.1)	**<0.001**
BMI, kg/m^2^ (95% CI)	451	26.6 (26.2–27.0)	26.6 (26.1–27.0)	26.7 (26.0–27.5)	0.695
Hypertension, N (%)	451	269 (59.6)	162 (57.2)	107 (63.7)	0.178
Systolic BP, mmHg, mean (95% CI)	442	146 (144–149)	145 (142–148)	149 (145–153)	0.12
Diastolic BP, mmHg, mean (95% CI)	441	84 (83–85)	84 (82–85)	85 (82–87)	0.398
Smoking, N (%)	451	169 (37.4)	98 (34.6)	71 (42.2)	0.106
Diabetes, N (%)	451	85 (18.8)	35 (12.4)	50 (29.8)	**<0.001**
LDL, mmol/L, mean (95% CI)	388	3.33 (3.24–3.43)	3.39 (3.27–3.52)	3.24 (3.08–3.39)	0.123
Imputed LDL, mmol/L, mean (95% CI)	451	3.33 (3.25–3.42)	3.38 (3.28–3.49)	3.25 (3.12–3.38)	0.138
hs-CRP, mg/L, mean (95% CI)	431	9.58 (7.76–11.4)	6.64 (4.60–8.68)	14.7 (11.3–18.0)	**<0.001**
Sedentary lifestyle, N (%)	424	73 (17.2)	27 (10.3)	46 (29.5)	**<0.001**
Previous stroke, N (%)	451	88 (19.5)	43 (15.2)	45 (26.8)	**0.003**
NIHSS score, mean (95% CI)	451	4.92 (4.44–5.41)	3.33 (2.89–3.77)	7.60 (6.67–8.54)	**<0.001**
s-IGFBP1, µg/L, mean (95% CI)	341	7.24 (6.36–8.13)	5.71 (5.16–6.27)	10.1 (7.87–12.4)	**0.015**
Insulin, microU/L, mean (95% CI)	430	15.5 (14.1–16.9)	14.0 (12.5–15.6)	18.0 (15.3–20.8)	**0.003**
Glucose, nmol/L, mean (95% CI)	427	6.37 (6.13–6.60)	6.00 (5.79–6.21)	6.99 (6.46–7.51)	**<0.001**
HOMA-IR, mean (95% CI)	413	4.58 (4.09–5.07)	3.93 (3.40–4.46)	5.69 (4.73–6.66)	**<0.001**

Values are presented as means and 95% CI or percentage fraction. The *p*-values are derived from Mann–Whitney U analysis (all continuous variables were non-normally distributed) and by Chi square analysis (categorical variables: sex, hypertension, smoking, diabetes). Statistically significant *p*-values (<0.05) are shown in bold. Abbreviations: BMI, body mass index; BP, blood pressure; hs-CRP, high-sensitivity C-reactive protein; HOMA-IR, homeostatic model assessment of insulin resistance; LDL, low-density lipoprotein; s-IGFBP1, serum levels of insulin-like growth factor-binding protein-1; NIHSS, National Institutes of Health Stroke Scale; mRS, Modified Rankin Scale; NA, not applicable.

**Table 5:** (**a**) Odds ratios (ORs) for poor functional outcome (mRS 3–6) after 7 years poststroke in patients with BMI 18.5–30. (**b**) Odds ratios (ORs) for poor functional outcome (mRS 3–6) after 7 years poststroke in patients with BMI > 25.

**(a)**						
**ORs for Poor Outcome (mRS 3–6) after 7 Years (BMI 18.5–30)**						
**Parameter**	**BMI 18.5–25 vs. 25–30 (ref = 1)**	***p***	**Per Log10 IGFBP-1 Increase**	***p***	**Per Log10 HOMA-IR Increase**	***p***
Unadjusted	2.22 (1.33–3.72)	**0.002**	2.96 (1.51–5.80)	**0.002**	3.57 (1.60–7.98)	**0.002**
Model 1	2.44 (1.43–4.14)	**0.001**	2.68 (1.35–5.32)	**0.005**	3.26 (1.44–7.39)	**0.005**
Model 2	2.32 (1.30–4.14)	**0.004**	4.67 (2.12–10.3)	**<0.001**	2.51 (1.03–6.12)	**0.043**
Model 2 + BMI 18.5–25	NA	NA	4.44 (2.00–9.89)	**<0.001**	3.68 (1.42–9.49)	**0.007**
Model 2 + IGFBP-1	2.21 (1.21–4.02)	**0.010**	NA	NA	3.63 (1.41–9.35)	**0.007**
Model 2 + HOMA-IR	2.84 (1.54–5.22)	**0.001**	5.67 (2.50–12.9)	**<0.001**	NA	NA
Model 2 + Diabetes	2.97 (1.59–5.55)	**0.001**	3.75 (1.67–8.41)	**0.001**	1.59 (0.61–4.19)	0.344
Model 2 + hs-CRP	2.34 (1.29–4.24)	**0.005**	4.47 (1.99–10.0)	**<0.001**	2.26 (0.91–5.65)	0.080
**(b)**						
**ORs for Poor Outcome (mRS 3–6) after 7 Years (BMI > 25)**						
**Parameter**	**BMI > 30 vs. 25–30 (ref = 1)**	***p***	**Per Log10 IGFBP–1 Increase**	***p***	**Per Log10 HOMA–IR Increase**	***p***
Unadjusted	2.44 (1.29–4.62)	**0.006**	2.01 (0.90–4.49)	0.088	5.34 (1.96–14.5)	**0.001**
Model 1	2.35 (1.23–4.51)	**0.01**	1.83 (0.80–4.18)	0.155	4.99 (1.81–13.8)	**0.002**
Model 2	2.25 (1.08–4.71)	**0.031**	3.81 (1.44–10.1)	**0.007**	3.06 (0.98–9.48)	0.053
Model 2 + BMI > 30	NA	NA	6.23 (2.12–18.32)	**0.001**	2.39 (0.74–7.79)	0.147
Model 2 + IGFBP-1	3.45 (1.53–7.76)	**0.003**	NA	NA	4.54 (1.39–14.8)	**0.012**
Model 2 + HOMA-IR	1.97 (0.92–4.22)	0.082	5.10 (1.82–14.3)	**0.002**	NA	NA
Model 2 + Diabetes	2.16 (1.02–4.58)	**0.045**	3.20 (1.19–8.60)	**0.021**	1.96 (0.57–6.70)	0.286
Model 2 + hs-CRP	2.12 (0.97–4.61)	0.058	3.87 (1.39–10.7)	**0.009**	3.95 (1.17–13.3)	**0.027**

(**a**) N = 271. (**b**) N = 202. Odds ratios (ORs) and 95% CIs were calculated using binary logistic regression. Values are presented as ORs with 95% CI and corresponding *p*-values. Statistically significant *p*-values (<0.05) are shown in bold. HOMA-IR, hs-CRP, and s-IGFBP1 have been Log10-transformed. Model 1 is adjusted for age and sex. Model 2, on top of Model 1: NIHSS, hypertension, current smoking and imputed serum LDL levels. Abbreviations: hs-CRP, high-sensitivity C-reactive protein; HOMA-IR, homeostatic model assessment of insulin resistance; LDL, low-density lipoprotein; s-IGFBP1, serum levels of insulin-like growth factor-binding protein-1; NIHSS, National Institutes of Health Stroke Scale; mRS, Modified Rankin Scale; NA, not applicable.

**Table 6:** Odds ratios (ORs) for poor outcome (mRS 3–6) after 7 years poststroke, for high and low IGFBP-1 in BMI categories.

BMI Category	n	OR for mRS 3–6, in *Low* IGFBP-1	OR for mRS 3–6, in *High* IGFBP-1
All BMI	330	NA	NA
BMI 18.5–25	128	**3.00 (1.35–6.68)**	**4.41 (2.00–9.73)**
BMI 25–30	144	ref = 1	**2.50 (1.14–5.51)**
BMI > 30	58	**3.53 (1.35–9.26)**	**4.67 (1.79–12.1)**

Values are presented as unadjusted odds ratios (ORs) with 95% CI. The ORs were calculated by binary regression. Patients are divided into six subgroups of high and low IGFBP-1 divided by median, and of BMI category. ORs are compared to the underlined reference category of participants (OR = 1, ref). This is defined as the group with the lowest amount of poor 7-year functional outcome, in this case n = 12 out of n = 72 (16.7%) in overweight (BMI 25–30) with low s-IGFBP-1. The medians of s-IGFBP-1 (µg/L) were for normal-weight 6.55, for overweight 4.54, and for the obese 2.92. Bold designates values with *p* < 0.05. Abbreviations: s-IGFBP-1, serum levels of insulin-like growth factor-binding protein-1; mRS, Modified Rankin Scale; NA, not applicable.

**Table 7:** Odds ratios (ORs) for poor outcome (mRS 3–6) after 7 years poststroke, for high and low *HOMA-IR* in BMI categories.

BMI Category	n	OR for mRS 3–6, in *Low* HOMA-IR	OR for mRS 3–6, in *High* HOMA-IR
All BMI	330	NA	NA
BMI 18.5–25	128	**3.00 (1.35–6.68)**	**4.41 (2.00–9.73)**
BMI 25–30	144	ref = 1	**2.50 (1.13–5.51)**
BMI > 30	58	2.63 (0.98–7.05)	**6.15 (2.36–16.1)**

Values are presented as unadjusted odds ratios (ORs) with 95% CI. The ORs are derived with binary regression. Patients are divided into six subgroups of high and low HOMA-IR divided by median, and of BMI category. ORs are compared to the underlined reference category of participants (OR = 1, ref). This is defined as the group with the lowest amount of poor 7-year functional outcome, in this case n = 12 out of n = 72 (16.7%) in overweight (BMI 25–30) with low HOMA-IR. The medians of HOMA-IR were for normal-weight 2.46, for overweight 3.18, and for the obese 5.25. Bold designates values with *p* < 0.05. Abbreviations: mRS, Modified Rankin Scale; NA, not applicable.

## Data Availability

The data presented in this study are available on reasonable request from the corresponding author. The data are not publicly available due to legal restrictions regarding privacy and ethical issues.

## Supplementary Materials

The following supporting information can be downloaded at: https://www.mdpi.com/article/10.3390/ijms25094931/s1. References [52,53,54,55,56] are cited in the supplementary materials.
