# Supplementary material for: A Nonlinear Relation between Body Mass Index and Long-Term Poststroke Functional Outcome—The Importance of Insulin Resistance, Inflammation, and Insulin-like Growth Factor-Binding Protein-1
Source: Int J Mol Sci. 2024 Apr 30;25(9):4931. doi: 10.3390/ijms25094931 (PMC11084577; doi:10.3390/ijms25094931)
Supplement: Supplementary file 1 [file ijms-25-04931-s001.zip › ijms-2930999-supplementary.pdf]

International Journal of *Molecular Sciences*

MDPI

A Nonlinear Relation between Body Mass Index and Long-Term Poststroke Functional Outcome—The Importance of Insulin Resistance, Inflammation, and Insulin-like Growth Factor-Binding Protein-1

Gustaf Gadd [1,2,*], Daniel Åberg [1,3], Alexander Wall [1,4], Henrik Zetterberg [5,6,7,8,9,10], Kaj Blennow [5,6,11,12], Katarina Jood [13,14], Christina Jern [15,16], Jörgen Isgaard [1,3], Johan Svensson [1,17] and N. David Åberg [1,2]

Supplementary materials

Contents:

- Details on description of study populations and clinical examination (items 1-4)
- Supplementary Table – Baseline parameters stratified for normal-weight, overweight and obese stroke patients.
- Supplementary Figure – flow chart
- Statistical note regarding comparison between adjusted and unadjusted effect measures
- References

Details on description of study populations and clinical examination

1. SAHLSIS: The Sahlgrenska Academy Study on Ischemic Stroke (SAHLSIS): The study population comprised patients of European descent who presented with first-ever or recurrent acute ischemic stroke (henceforth stroke) before reaching the age of 70 years (n=600). The patients were consecutively recruited between 1998 and 2003 at four stroke units in Western Sweden (1, 2). The SAHLSIS study was originally an observational cohort study with prospective collection of serum samples and functional assessments [1, 3]. Population controls (n=600), not affected by cardiovascular disease, were included as previously reported [1, 2]. During the long-term follow-ups some of the ensuing reports [4] have not used the control arm data, which is also the case for this study. The present study is thus a prospective longitudinal observational study on ischemic stroke.
2. Clinical definitions and descriptions: Stroke was defined as an episode of focal neurological deficits with acute onset and lasting for >24 hours or until death, with no apparent non-vascular cause, and no signs of primary hemorrhage on brain imaging. The clinical rate of thrombolysis in 1998-2003 was very low, in this material only 1.3% (N=5), and there were no patients having undergone thrombectomy. All patients underwent ECG and neuroimaging with computed tomography (CT) and/or magnetic resonance imaging (MRI). Extracranial carotid and vertebral duplex ultrasound, MR angiography, catheter angiography, transcranial Doppler ultrasound, and transthoracic and/or transesophageal echo-cardiography were performed when clinically indicated. The patients received standard rehabilitation at the Stroke unit and later at primary care or nursing homes. This rehabilitation is always adapted to the individual patient's needs and might differ depending on whether the brain injury involves speech, motor, visual or cognitive dysfunction. In routine praxis, most stroke patients with handicaps receive such therapy in Sweden [1]. However, the specific individual rehabilitation was not recorded as the original aim of the SAHLSIS study was to

study genetic polymorphisms in the fibrinolytic system as found in the first two published articles.

3. Patient examination: Functional outcome at 3 months, 2 years, and 7 years after index stroke was assessed using the mRS scale. Over the years, these were performed by one neurology specialist physician at baseline and at the 3-month follow-up. At the 2-year and 7-year follow-ups a research nurse, trained in stroke medicine by the research specialist physician, assessed functional outcome. Stroke severity was assessed at the inpatient clinic while all other functional assessments including mRS were performed outpatient on follow-up occasions outside the hospital ward. Information on the subjects' vascular risk factors was collected as described previously [1]. Hypertension was defined by pharmacological treatment for hypertension, systolic blood pressure ≥160 mm Hg, and/or diastolic blood pressure ≥90mm Hg. Diabetes mellitus was defined by diet or pharmacological treatment, fasting plasma glucose ≥7.0 mmol/L or fasting blood glucose ≥6.1 mmol/L. Smoking habits were coded as current, never or former. In the present study, this was dichotomized into current smoker or non-smoker (which included never- and former smokers). Concentrations of low-density lipoprotein (LDL, mmol/L) were measured as before [1, 5]. The missing values (N=63, Table 1) were imputed using the mean of the baseline LDL levels to have complete LDL datasets when used as a covariate in the regression models. The pre-stroke PA level for the last 12 months before the index stroke was assessed in a questionnaire using a self-report scale, with four levels of PA, the Saltin-Grimby Physical Activity Scale (SGPALS), in which PA1 represents a sedentary lifestyle, and PA2 to PA4 representing progressively higher levels of PA.
4. Scandinavian Stroke Scale (SSS) and NIHSS: Maximum stroke severity within the first 10 days after the stroke was scored using the Scandinavian Stroke Scale (SSS). SSS is a 58-point scale, in which a higher score represents better functionality. The SSS is highly (but inversely) correlated to the National Institutes of Health Stroke Scale (NIHSS). In this study, global SSS scores were transformed to NIHSS scores using a conversion algorithm [6], as described in the main text. Functional outcome 3 months, 2 years and 7 years after stroke was assessed according to the modified Rankin Scale (mRS) [7], later modified to seven steps (0-6) [8]. The mRS 0-2 scores represent functional independence (good outcome), whereas scores 3-6 represent functional dependence (poor outcome), of which mRS 6 designates death.

Supplementary Table S1. Death after 3 months, 2 years and 7 years

**Death – time point**	**n**	**p (3-group)**	**A. Normal-weight (BMI 18.5-25)**	**p, A vs. B**	**B. Overweight (BMI 25-30)**	**p, B vs. C**	**C. Obese (BMI>30)**	**p, A vs. C**
Death after 3 months [n / total n, (%)]	2/432 (0.5)	0.346	0/172 (0)	0.338	1/187 (0.5)	0.490	1/73 (1.3)	0.125
Death after 2 years [n / total n, (%)]	17/449 (3.8)	0.218	10/180 (5.6)	0.081	4/191 (2.1)	0.389	3/78 (3.8)	0.566
Death after 7 years [n / total n, (%)]	79/451 (17.5)	0.005	42/180 (23.3)	**0.001**	21/193 (10.9)	**0.037**	16/78 (20.5)	0.620
Death after 7 years - AIS [n / total n, (%)]	12/451 (2.7)	0.438	6/180 (3.3)	0.263	3/193 (1.6)	0.246	3/78 (3.8)	0.937
Death after 7 years - HS [n / total n, (%)]	3/451 (0.67)	0.569	2/180 (1.1)	0.522	1/193 (0.5)	0.524	0/78 (0)	0.352
Death after 7 years - CVD [n / total n, (%)]	20/451 (4.4)	0.437	9/180 (5.0)	0.353	6/193 (3.1)	0.212	5/78 (6.4)	0.646
Death after 7 years – other [n / total n, (%)]	44/451 (9.8)	**0.032**	25/180 (13.4)	**0.009**	11/193 (5.7)	0.25	8/78 (10.3)	0.363

Numbers (n) of patients are shown for death (mRS 6), total n, and percentage fraction. The p-values are derived from Chi-square analysis, the first column shows all three groups compared, and in the following columns, specific comparisons, as indicated. Statistically significant p-values (<0.05) are shown in bold. Abbreviations: AIS; acute ischemic stroke, HS; hemorrhagic stroke, BMI; body mass index, CVD; cardiovascular disease [any vascular or cardiac cause, but not stroke].

Supplementary Table S2. Sensitivity analysis. ORs for poor outcome (mRS 3-6) after 7 years

Parameter	BMI 18.5-25 vs 25-30 (ref=1)	p	BMI>30 vs 25-30 (ref=1)	P
Unadjusted	2.22 (1.33-3.72)	**0.002**	2.44 (1.29-4.62)	**0.006**
Model 2	2.32 (1.30-4.14)	**0.004**	2.25 (1.08-4.71)	**0.031**
Model 2 + mRS 6 (Death, N=79)	1.23 (0.58-2.62)	0.587	2.23 (0.94-5.27)	0.068
Model 2 + previous stroke (N=88)	2.43 (1.34-4.41)	**0.003**	2.32 (1.08-4.98)	**0.030**
Model 2 + sedentary lifestyle (N=73)	3.01 (1.58-5.74)	**0.001**	1.72 (0.74-4.06)	0.211

N = 271 for left column and N=202 for right column. Odds ratios (ORs) and 95% CIs were calculated using binary logistic regression. Values are presented as ORs with 95% CI and corresponding p-values. Statistically significant p-values (<0.05) are shown in bold. Model 2: Adjusted for age, sex, NIHSS, hypertension, current smoking and imputed serum LDL levels. Abbreviations: NIHSS, National Institutes of Health Stroke Scale; mRS, Modified Rankin Scale**.**

Supplementary Table S3. Baseline parameters for patients with both HOMA-IR and s-IGFB1 at 7-year poststroke stratified for normal-weight, overweight and obese stroke patients.

**Variable**	**N**	**A. Normal-weight (BMI 18.5-25)**	**p-value A vs. B**	**B. Overweight (BMI 25-30)**	**p-value B vs. C**	**C. Obese (BMI<30)**	**p-value A vs. C**
All patients (N, %)	330	128 (100)	NA	144 (100)	NA	58 (100)	NA
Females (N, %)	122	52 (40.6)	0.108	45 (31.3)	0.110	25 (43.1)	0.752
Males (N, %)	208	76 (59.4)	0.108	99 (68.8)	0.110	33 (56.9)	0.752
Age, years (95% CI)	330	55.8 (53.8-57.7)	0.224	57.9 (56.5-59.3)	0.728	58.3 (56.2-60.5)	0.227
BMI , kg/m^2 (95% CI)	330	23.1 (22.8-23.3)	**<0.001**	27.2 (26.9-27.4)	**<0.001**	33.4 (32.6-34.3)	**<0.001**
Hypertension (N, %)	330	68 (53.1)	0.149	89 (61.8)	**0.008**	47 (81.0)	**<0.001**
Systolic BP, mmHg, mean (95% CI)	325	143 (138-148)	0.112	146 (143-150)	0.946	147 (141-154)	0.172
Diastolic BP, mmHg, mean (95% CI)	325	82 (80-85)	0.112	84 (82-86)	0.91	84 (81-87)	0.215

Smoking (N, %)	330	57 (44.5)	0.077	49 (34.0)	0.864	19 (32.8)	0.132
Diabetes (N, %)	330	13 (10.2)	**0.007**	32 (22.2)	0.290	17 (29.3)	**0.001**
LDL, mmol/L, mean (95% CI)	294	3.18 (3.01-3.35)	**0.011**	3.52 (3.35-3.69)	0.535	3.41 (3.07-3.74)	0.274
Imputed LDL, mmol/L, mean (95% CI)	330	3.19 (3.04-3.35)	**0.007**	3.50 (3.34-3.65)	0.492	3.39 (3.11-3.68)	0.185
hs-CRP, mg/L, mean (95% CI)	329	12.4 (7.69-17.1)	0.963	8.05 (5.40-10.7)	**0.030**	9.52 (5.48-13.6)	0.059
Sedentary lifestyle (N/tot N, %)	311	11 (8.59)	0.258	18 (12.5)	**<0.001**	20 (34.5)	**<0.001**
Previous stroke (N/tot N, %)	330	24/128 (18.8)	0.401	33/144 (22.9)	0.939	13/58 (22.4)	0.564
NIHSS	330	4.94 (3.99-5.89)	0.750	4.57 (3.75-5.38)	0.217	5.13 (3.85-6.41)	0.164
s-IGFBP1, μg/L, mean (95% CI)	330	8.61 (7.03-10.2)	**0.023**	7.06 (5.60-8.53)	**0.014**	4.42 (3.40-5.44)	**<0.001**
Insulin, microU/L, mean (95% CI)	330	13.6 (10.8-16.4)	**0.003**	14.9 (13.1-16.7)	**<0.001**	23.0 (17.5-28.5)	**<0.001**
Glucose, nmol/L, mean (95% CI)	330	5.99 (5.55-6.43)	**0.007**	6.74 (6.29-7.20)	0.271	6.94 (6.23-7.65)	**0.001**
HOMA-IR, mean (95% CI)	330	3.76 (2.91-4.61)	**<0.001**	4.76 (3.88-5.64)	**<0.001**	6.91 (5.39-8.43)	**<0.001**

Baseline parameters stratified for normal-weight, overweight and obese stroke patients with both s-IGFBP1 and HOMA-IR measurements available (n=330), thus used for Table 6. Values are presented as means and 95% CI or percentage fraction. The p-values are based on Mann-Whitney U analysis (all continuous variables were non-normally distributed) and by Chi square analysis (categorical variables: sex, hypertension, smoking, diabetes). Statistically significant p-values (<0.05) are shown in bold. Abbreviations: BMI, body mass index; BP, blood pressure; hs-CRP, high-sensitivity C-reactive protein; HOMA-IR, homeostatic model assessment of insulin resistance; LDL, Low-density lipoprotein; s-IGFBP1, serum levels of insulin-like growth factor binding protein 1; NIHSS, National Institutes of Health Stroke Scale.

Supplementary Figure S1- flow chart

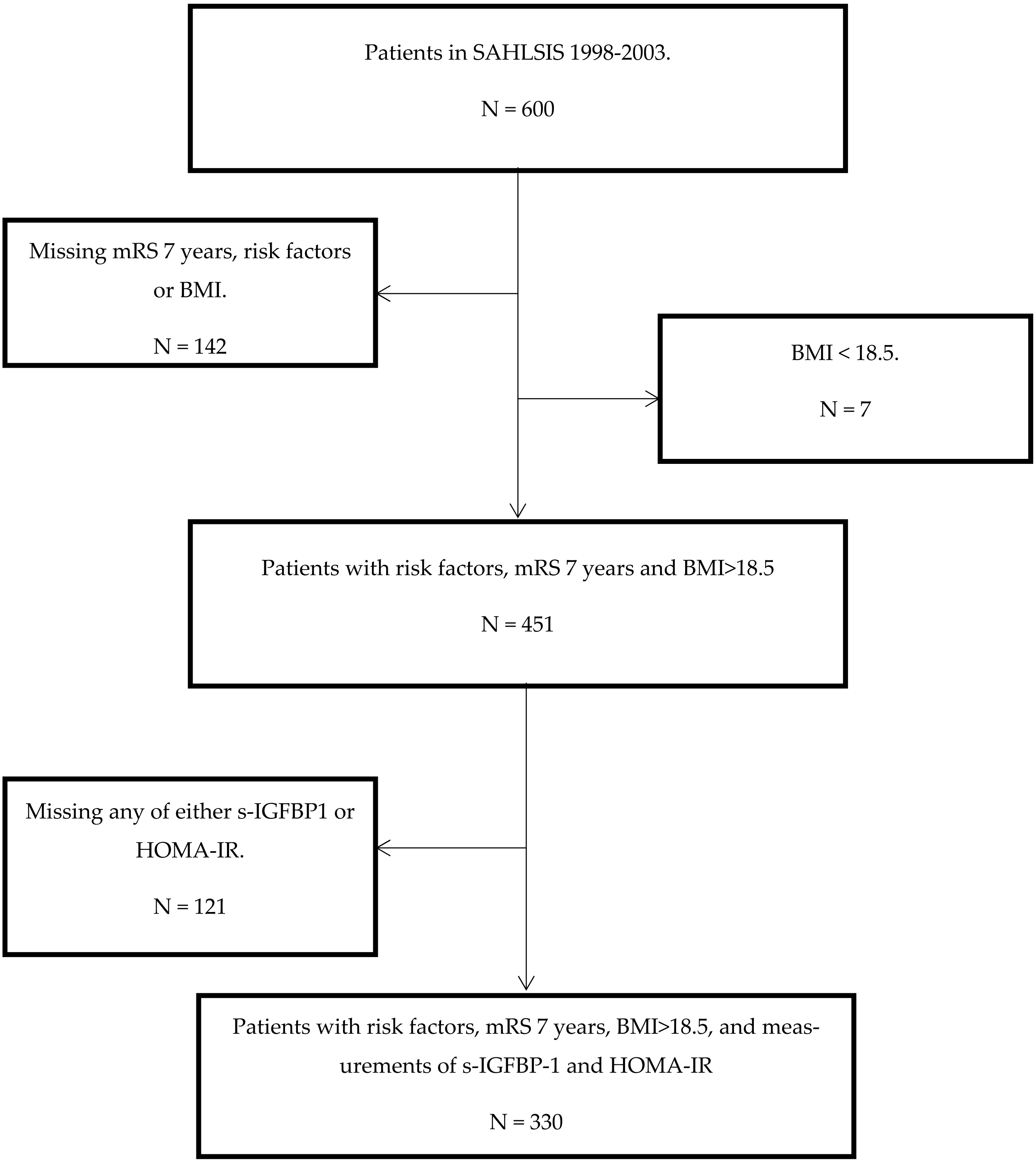

Flowchart showing the numbers of included subjects, as well as the number of excluded patients for different subgroups.

Statistical note regarding comparison between adjusted and unadjusted effect measures

To assess how much the covariate adjustment affected the effect measure of X with respect to the binary outcome (Y) we consider the relative effect of X on risk of Y, i.e.

$$\exp(b)-1$$

where b denotes the beta-value for X in the logistic regression model

$logit(p(Y|X))=a+bX$, and $(\exp(b)-1)$ 100% describes how much the risk for Y changes when X changes by 1 unit (in percent).

Denoting b = unadjusted beta value, b′ = adjusted beta-value for X, the relative difference between unadjusted and adjusted effect size is given by

$$\frac{(\exp(b)-1)-(\exp(b')-1)}{\exp(b)-1} = \frac{\exp(b)-\exp(b')}{\exp(b)-1}$$

For small effect size $b \ll 1$, we have $\exp(b) \sim 1+b$, and the relative difference due to adjustment reads

$$\frac{(\exp(b)-1)-(\exp(b')-1)}{\exp(b)-1} = \frac{b-b'}{b}$$

i.e. it is equal to the relative difference on beta-value level.

References:

1. Jood K, Ladenvall C, Rosengren A, Blomstrand C, Jern C. Family history in ischemic stroke before 70 years of age: the Sahlgrenska Academy Study on Ischemic Stroke. Stroke. 2005;36(7):1383-7.
2. Olsson S, Jood K, Blomstrand C, Jern C. Genetic variation on chromosome 9p21 shows association with the ischaemic stroke subtype large-vessel disease in a Swedish sample aged≤ 70. Eur J Neurol. 2011;18(2):365-7.
3. Jood K, Ladenvall P, Tjarnlund-Wolf A, Ladenvall C, Andersson M, Nilsson S, et al. Fibrinolytic gene polymorphism and ischemic stroke. Stroke. 2005;36(10):2077-81.
4. Aberg ND, Aberg D, Jood K, Nilsson M, Blomstrand C, Kuhn HG, et al. Altered levels of circulating insulin-like growth factor I (IGF-I) following ischemic stroke are associated with outcome - a prospective observational study. BMC Neurol. 2018;18(1):106.
5. Olsson S, Jood K, Blomstrand C, Jern C. Genetic variation on chromosome 9p21 shows association with the ischaemic stroke subtype large-vessel disease in a Swedish sample aged <= 70. Eur J Neurol. 2011;18(2):365-7.
6. Gray LJ, Ali M, Lyden PD, Bath PM, Virtual International Stroke Trials Archive C. Interconversion of the National Institutes of Health Stroke Scale and Scandinavian Stroke Scale in acute stroke. J Stroke Cerebrovasc Dis. 2009;18(6):466-8.
7. Rankin J. Cerebral vascular accidents in patients over the age of 60. II. Prognosis. Scott Med J. 1957;2(5):200-15.
8. Banks JL, Marotta CA. Outcomes validity and reliability of the modified Rankin scale: implications for stroke clinical trials: a literature review and synthesis. Stroke. 2007;38(3):1091-6.
